# How to achieve high-quality development of SRDI enterprises—A study of the TOE framework-based configuration

**DOI:** 10.1371/journal.pone.0304688

**Published:** 2024-06-03

**Authors:** Zhihong Zhang, Hua Feng, Lulu Wang, Lingyun Yang

**Affiliations:** School of Accountancy, Shandong University of Finance and Economics, Jinan, China; IBS Hyderabad: ICFAI Business School, INDIA

## Abstract

The high-quality development of SRDI enterprises is crucial for China to overcome critical technological bottlenecks and thereby achieve technological independence and strength. However, the factors driving the high-quality development of SRDI enterprises are not isolated elements, but rather a complex system of interconnected antecedents. This study employs the TOE framework and fuzzy set Qualitative Comparative Analysis (fsQCA) with 141 Chinese SRDI “little giant” listed companies as samples to explore how various factors contribute to their high-quality development. The findings indicate: (1) No single factor is necessary for SRDI enterprises’ high-quality development. (2) It is the synergy of multiple factors, in various combinations, that drives their high-quality development. (3) Technological innovation plays a key role in these pathways; SRDI enterprises should leverage their resources and capabilities for a synergistic technology-organization-environment match, selecting the most suitable development path. The results of this study not only enrich our understanding of the factors influencing SRDI enterprises’ high-quality development but also offer insights for both the enterprises and government policy-making.

## 1. Introduction

Small and medium-sized enterprises are a pivotal force in China’s economic and social development, playing a crucial role in stabilizing the national economic growth rate and boosting economic vitality [1]. SRDI enterprises are defined as small and medium-sized enterprises with characteristics of specialization, refinement, distinctiveness, and innovation. Focusing on specific niche markets, they offer high-quality products and services with unique advantages, aiming for a leading position in market share and innovation within their niches [2]. In the context of economic deglobalization, the competition between nations has evolved from trade disputes to battles over technological prowess. The ongoing China-US trade friction has escalated the technological rivalry between China and leading developed countries. However, in comparison with developed nations, China faces gaps in developing its innovation ecosystem, including infrastructure, professional talent, and industry support. This leaves China at a disadvantage in key technological areas, facing the challenge of technological dependency. Therefore, overcoming the comprehensive technological blockade by developed countries and addressing the “stuck neck” issue is crucial for China to achieve high-quality economic development. Enterprises, serving as the foundation of macroeconomic development, are key micro-elements for achieving economic high-quality development. Promoting the high-quality development of enterprises is essential for advancing the high-quality development of the macroeconomy. SRDI enterprises, with their technological breakthroughs and barriers, offer a “Chinese solution” to the “stuck neck” issue, bolstering the security, stability, and resilience of our industrial and supply chains, and aiding China’s pursuit of high-quality economic development. This also serves as a practical reference for other emerging markets striving for key technological breakthroughs.

The niche market-focused development model for SMEs has seen vigorous growth in China, originating from Western developed countries. In 1992, Hermann Simon [3] identified German SMEs leading in niche markets, despite their small size and lesser-known brands, as “Hidden Champions.” Germany’s “Hidden Champions” have thrived with support from the government, associations, and financial systems, alongside their own focus on business and globalization strategies, securing a leading position in global niche markets. The niche market-focused SME development model subsequently gained popularity in Europe and influenced Asian regions, including Japan and China. Japanese and Chinese SMEs have since realigned their development strategies towards niche markets. Japan has bolstered its SMEs’ international competitiveness by fostering “high-niche enterprises” through policies, funding, and recognition, promoting a focus on segmented markets, core technologies, and global industry chains. The Chinese government’s focus on SRDI enterprises started with the 2011 “Twelfth Five-Year Plan for the Growth of SMEs” by the Ministry of Industry and Information Technology of China, introducing and prioritizing the “specialized, refined, distinctive, and innovational” concept. Subsequently, the Chinese government enacted several policies favoring SRDI enterprise development. With significant government support, Chinese SRDI enterprises have experienced rapid growth. By December 2023, China has successfully cultivated more than 10,000 national-level SRDI “little giant” enterprises, significantly accelerating the high-quality development of the Chinese economy.

Existing studies on SRDI enterprises primarily examine the influence of individual factors on their development, overlooking the comprehensive pathways and driving mechanisms essential for high-quality development. It should be noted that the high-quality development of SRDI enterprises encompasses a complex system engineering process, where the interplay among various factors produces a cumulative effect. Employing linear regression to analyze the impact of a single factor does not align with the multifaceted development context of SRDI enterprises. This approach fails to adequately uncover the pathways to high-quality development and the intricate interplay among various factors, thus complicating the elucidation of complex causal relationships among diverse factors and configurations. Therefore, this study adopts an integrative approach to develop a Technology-Organization-Environment (TOE) theoretical framework. It focuses on 141 SRDI “little giant” companies listed in China in 2020, utilizing Fuzzy Set Qualitative Comparative Analysis (fsQCA). This method examines the key drivers of SRDI enterprises’ high-quality growth and the intricate configurations resulting from the interactions among these drivers, aiming to uncover pathways towards achieving such growth.

The incremental contributions of this study are reflected in three key areas: Firstly, prior research primarily focuses either on the impact of external factors—like fiscal subsidies [4], innovation policy [5], tax incentives [6] and the business environment [7]—or on internal factors—such as digital transformation [8] and entrepreneur characteristics [9]—on the development of SRDI enterprises. However, SRDI enterprises’ development is influenced by a combination of both internal and external factors. Analyzing the impact of isolated factors on the high-quality development of SRDI enterprises may introduce bias. This study adopts a holistic approach, integrating both internal and external factors into its research framework, significantly contributing to a comprehensive understanding of the factors influencing SRDI enterprises’ development.

Secondly, traditional research often employs regression analysis methods [5, 6] to examine the net effects of single factors on SRDI enterprises’ development. However, the reality is that a diverse range of factors, often working in synergy, affect SRDI enterprises’ development. Examining the impact of a single factor on SRDI enterprises’ development lacks scientific rigor. Therefore, from a systems perspective, this study utilizes the fuzzy-set qualitative comparative analysis method to systematically assess the combined effects of multiple factors, thereby enhancing the research’s effectiveness.

Thirdly, numerous studies [10, 11] approach from a theoretical angle, discussing ways SRDI enterprises can improve their development quality. The conclusions of these studies are primarily derived theoretically. Using 141 Chinese SRDI enterprises as samples, this study explores pathways to high-quality development through micro-level empirical data, offering insights for SRDI enterprises and guiding government support policies. This approach not only provides high-quality development ideas for SRDI enterprises but also offers a realistic basis for the government to formulate appropriate support policies for SRDI enterprises.

The structure of this study is as follows. Section 2 presents the literature review. Section 3 details the model construction. Section 4 outlines the research design, including methods, variable definitions, and data selection and calibration. Section 5 analyzes the empirical results in detail. Finally, Section 6 summarizes the conclusions and discusses future research directions.

## 2. Literature review

Current scholarly research on enterprise high-quality development is extensive yet lacks consensus. Atta et al. [12] argued that high-quality development demands the provision of superior products and services, focusing on the efficiency of creating economic and social value, and enhancing corporate competitiveness and growth potential. Li et al. [13] posited that high-quality enterprise development relies on technology, resource integration for competitive advantage, and the harmonization of economic, social, and environmental benefits. Wang et al. [14] suggested that alongside resource conservation and efficiency, high-quality development should incorporate environmentally friendly governance, promoting sustainable development. In summary, high-quality development signifies enterprises’ achievement of superior and distinguished development quality [15].

Based on enterprise growth theory, SRDI enterprises’ high-quality development necessitates the continuous accumulation of knowledge and resources for competitive advantages [16]. The essence of SRDI enterprises’ high-quality development is to secure key technologies via continuous innovation, achieving dominance in niche markets. Implementing a “Niche Strategy” encourages the development of essential technologies for various applications, addresses real-world challenges, and expands into new markets beyond the constraints of niche market sizes. This approach fosters a sustainable development cycle powered by technological innovation.

Studies on the high-quality development of SRDI enterprises have concentrated on both macro-policy and micro-enterprise factors. Indeed, SRDI enterprises’ high-quality development is shaped by diverse and complex factors from both macro and micro levels, with their impact varying across different contexts. This study employs the TOE framework to examine both macro and micro factors influencing SRDI enterprises, reviewing the literature on their development impacts from technological, organizational, and environmental perspectives.

On the technological level, Tavalaei et al. [17] found that technological innovation is crucial for maintaining competitive advantage, improving product quality, and fostering high-quality development. Song et al. [18] explored green technology’s impact on resource-based enterprises, revealing it boosts total factor productivity by enhancing unit labor productivity. Cheng et al. [19] examined how digital technology elevates enterprise development quality through improved human capital structure and working capital turnover efficiency. Zhou et al. [20] discovered that hard technology innovation drives leapfrog growth in SRDI enterprises, with digitalization amplifying its positive effects. Wu et al. [21] utilized a bidirectional fixed-effect model to explore the effects of digital transformation on high-quality enterprise development. The study revealed that digital transformation could foster high-quality development in enterprises by enhancing information transparency and technological innovation. Tung and Baird [22] argued that technological innovation broadens enterprise development perspectives, encouraging the creation of new products within environmental regulatory frameworks for profit. Despite the associated cost increases, the benefits of new technologies can offset these costs, boosting the incentive for environmental performance improvement and sustainable development.

On the organizational level, Xie et al. [23] explored how informal board practices can address formal institutional shortcomings, finding they enhance corporate governance and high-quality development. Bai et al. [24] examined the impact of ownership structure on state-owned enterprises, revealing it significantly influences high-quality development through management and operational improvements. Xue et al. [25] found that corporate social responsibility contributes to high-quality development by fostering green innovation and refining governance. Jiang et al. [26] demonstrated that disclosing environmental information boosts high-quality development by enhancing intellectual capital.Kaushal et al [27] discovered that the strategic choices of enterprises critically influence their development. Based on Michael Porter’s differentiation strategy, enterprises offering unique professional products and services can swiftly build brand recognition, foster brand loyalty, and lessen customer price sensitivity, thereby enhancing product and service profitability. When competing with similar products, the differentiation strategy enables enterprises to sustain stable performance and elevate development quality. Bai et al. [28], using Chinese advanced manufacturing enterprises as samples, revealed that a robust entrepreneurial spirit fosters a culture that tolerates failure, encourages innovation, stimulates employee creativity, boosts innovation activities, significantly enhances organizational performance, and leads to high-quality enterprise development.

On the environmental level, Cao et al. [5] examined how China’s innovation policies stimulate SRDI enterprises’ innovation quality, highlighting government innovation funds’ role in easing financing constraints and offsetting innovation externalities. Dun and Mao [29] identified the business environment as a crucial factor in SRDI enterprises’ high-quality development through a resource allocation lens. Dong et al. [30] demonstrated that a conducive business environment is key to high-quality development, sparking innovation vitality and enhancing innovation performance. Kong et al.’s study on China’s anti-monopoly policy revealed that industry competition boosts investment efficiency and innovation performance, thereby improving total factor productivity [31]. Zhang et al. [4] empirically examined how government support policies for private enterprises enhance the development of SRDI enterprises. They found that supply-side policies effectively alleviate SRDI enterprises’ resource dilemmas; demand-side policies stabilize their market expectations; and environmental policies improve their business environment. Cao and Xia [6] explored the effects of industrial policy implementation on SRDI enterprises’ total factor productivity. They discovered that financial subsidies, tax incentives, and other policies significantly ease SRDI enterprises’ financing constraints, thus boosting their total factor productivity.

Despite significant research on the connotation and factors influencing high-quality enterprise development, shortcomings remain: First, while enterprise high-quality development is a hot academic topic, studies focusing on SRDI enterprises are scarce. Second, there is a gap in systematic research on factors influencing SRDI enterprises’ high-quality development and their development pathways. SRDI enterprises’ high-quality development results from complex interactions among multiple elements, not just their simple aggregation. The oversight of the holistic nature of influencing factors limits research in identifying their interplay, complicating the understanding of SRDI enterprises’ development dynamics. Given these gaps, our study seeks to answer: What interactions promote SRDI enterprises’ high-quality development? Which elements are pivotal in their development paths? Is there a “multiple concurrent” phenomenon in their development?

## 3. Model construction

The TOE framework was first proposed by Tornatizky and Fleischer in 1990, examining its impact on organizational technology adoption across the dimensions of technology, organization, and environment. Through years of development, the framework’s scope has expanded, embracing a wealthier connotation and broader application domains. Amid the critical technological “stuck neck” scenarios, Chinese SRDI enterprises grapple with challenges such as technological innovation dilemmas, intricate operational management, and dynamic development environments. Technological innovation, as emphasized by the TOE framework, stands as the bedrock for SRDI enterprises, crucial for enhancing their development quality [32]. Overcoming the technical obstacles necessitates SRDI enterprises to possess the agility to adapt swiftly to environmental fluctuations, with organizational conditions at the center of the TOE framework’s focus. Market competition, policies, and regulations represent external environmental factors highlighted by the TOE framework, underscoring the significance of a conducive market, proactive government, and other external players for the development of SRDI enterprises. The interconnectedness of technology, organization, and environment within the TOE framework collectively shapes the development quality of SRDI enterprises, aligning closely with the research focus of this study.

### 3.1 Technological dimension

(1) Innovation capacity

Innovation capability (IC) is defined as an enterprise’s ability to continuously adapt and improve through technological innovations, securing new technologies, products, and services for a competitive edge in the market [33]. For SRDI small and medium-sized enterprises, innovation capability signifies their technological innovation level and is crucial for maintaining a leadership position in niche markets. Consequently, this study integrates innovation capability as a technical element within its research framework.

Schumpeter’s endogenous growth theory posits that technological progress, stemming from innovation, plays a crucial role in driving enterprise growth. Firstly, technological innovation transforms production modes, enabling smarter, automated processes and reducing labor input per output unit. Labor substitution through technological innovation enables cost reduction and higher efficiency in production, boosting overall productivity. Second, by facilitating the organization, collection, storage, and processing of extensive data, technological innovation enhances customer information accuracy and market response prediction, boosting marketing effectiveness [34]. Thirdly, technological innovation fosters internal data transparency, reduces communication costs, strengthens collaboration, and encourages a shift towards a more efficient, flat management model, thereby improving decision-making and productivity. As pioneers among Chinese SMEs, SRDI enterprises encounter resource constraints in niche markets. Continuous innovation capability enhancement is essential for securing a first-mover advantage, exploring new niche markets, and overcoming the specialization lock-in challenge.

(2) Digital transformation

Digital transformation (DT) is defined as the enhancement of enterprise production and management via digital technologies like big data, cloud computing, and artificial intelligence. This process involves altering the activities within an enterprise’s value creation process [35]. In the booming era of digital technology, how SRDI enterprises utilize digital technologies directly influences their ability to stay current, satisfy emerging niche market demands, and sustain their development. Consequently, this study defines the application level of digital technology in enterprises as digital transformation and integrates it as a key technological factor within the research framework.

Based on dynamic capability theory, digital transformation plays a vital role in empowering enterprises to gain a competitive edge in the market through digital enablement. In the dynamic landscape of competition, organizations are required to continually amass and cultivate their internal resources, technological capabilities, and overall competencies to effectively navigate through rapidly evolving market dynamics and position themselves competitively [36]. Essential facets for achieving competitive edges in such dynamic contexts involve product supply, integrative effort, and resource exchange. Through digitization empowerment, SRDI enterprises can streamline their business operations, improve efficiency in supply-demand coordination, element integration, and transaction circulation. This transformation allows enterprises to break through organizational boundaries, access valuable knowledge resources, capture real-time market insights, and transition from relying on past experiences for decision-making to proactive and anticipatory sensing [37]. By conducting accurate assessments and dynamic optimizations of core resources like enterprise strategy, products, technology, and services, SRDI enterprises can better cater to customer value needs. This enables them to elevate business performance and operational efficiency in the ever-changing market landscape [38], solidify their development advantages, foster value co-creation within specialized industry segments, consolidate their relative strengths, and establish themselves as leading players in their fields [39].

### 3.2 Organizational level

(1) Corporate social responsibility

Corporate social responsibility (CSR) originates from the enterprise’s subjectivity, focusing on maintaining good organizational performance, being responsible to employees and local communities, and promoting positive social public values [40]. The fulfillment of corporate social responsibility by SRDI enterprises, and the extent to which they do so, reflects the organizational capability and management level of these enterprises. Corporate social responsibility is incorporated as an organizational element within the research framework.

According to stakeholder theory, enterprises that fulfill their social responsibilities effectively maintain legitimacy, reduce risks, enhance reputation, and build competitiveness [41]. This not only serves stakeholders’ interests but also boosts profitability, marking a vital chance to elevate both economic and social values [42]. SRDI enterprises, which adopt a specialization strategy in niche markets, face resource constraints that can limit their ability to achieve scale economies [43]. By fulfilling social responsibilities, SRDI enterprises can broaden their niche markets, increase product coverage, reduce dependency on dominant firms, enhance operational autonomy and negotiation power, achieve higher returns, secure market leadership, and create industry spillover effects.

(2) Managerial stock ownership

Managerial stock ownership (MSO) is an equity compensation system that motivates management to align their work behavior with the enterprise’s long-term development strategy, granting them conditional ownership of shares [44]. Drawing on agency theory, managerial stock ownership can mitigate the enterprise’s agency problems and improve the rigor of its organizational management decisions. Most SRDI enterprises, typically small and medium-sized, face pronounced agency problems due to their evolving organizational and equity structures. Managerial stock ownership serves as a gauge for assessing the resolution of these issues and the overall state of organizational management. Managerial stock ownership is thus incorporated as an organizational element within the research framework.

Resource orchestration theory argues that a company’s internal resources are crucial for maintaining its competitive advantage. Continuously adjusting internal resources in response to the external environment is vital for building a company’s core competitiveness [45]. Management shareholding can effectively mitigate agency problems, boost management and operational efficiency, and enhance enterprise value [46]. Most SRDI enterprises in China are private entities with initially limited resources [8]. Management shareholding not only invigorates management but also fosters a customer-oriented approach in niche markets. It leads to dynamic adjustment of resources and capabilities based on customer needs, through refined management and process transformation. This boosts customer satisfaction, tightens cooperation, deepens industrial chain integration, showcases management and technology spillover effects, and strengthens competitive advantages.

### 3.3 Environmental dimension

(1) Market competition

Market competition(MC) involves similar economic actors enhancing their strength and excluding competitors’ actions for self-interest [47]. Within fierce market competition, SRDI enterprises need to target niche markets, offer superior products, and cultivate strong brand images to elevate their development quality. Market competition serves as a crucial external factor for SRDI enterprises’ development, thus is incorporated into our research framework.

Competitive advantage theory suggests that enterprises can achieve competitive advantages through cost leadership, differentiation, and focus strategies. SRDI enterprises’ competitive strategies blend focus on “specialization and refinement” with differentiation through “uniqueness and innovation”. Despite gaining a strategic edge in their target markets, SRDI enterprises are compelled to adapt to disruptive innovations and intense competition, particularly in critical technology areas [48]. Implementing a “T-shaped strategy” that focuses on deepening technology expertise vertically and expanding application scenarios horizontally, along with penetrating niche markets, helps them maintain a stable and advantageous position, leading to “passive efficiency gains”.

(2) Business environment

The business environment (BE) encompasses all external factors, including administrative, market, legal, and cultural conditions faced by market entities during entry, operation, and exit processes [49]. A positive business environment, influencing regional market activities, can notably lower market institutional costs, ensure equitable access to production factors for all market entities, and facilitate their market-based allocation. Hence, it is integrated into the study framework.

A conducive business environment enhances market efficiency. It mitigates environmental uncertainty, sharpens market expectation accuracy, aids in risk avoidance, cuts non-productive spending, and boosts vitality and competitiveness in market-oriented operations [50]. SRDI enterprises aiming to overcome their specialization “lock-in” require a favorable development environment. This is crucial for regional enterprise development, particularly for SMEs [51]. Despite their robust technology and management, SRDI enterprises’ development sustainability may be limited in a less favorable business environment [52].

### 3.4 Construction of high-quality development driver model for SRDI enterprises

**Fig 1:**
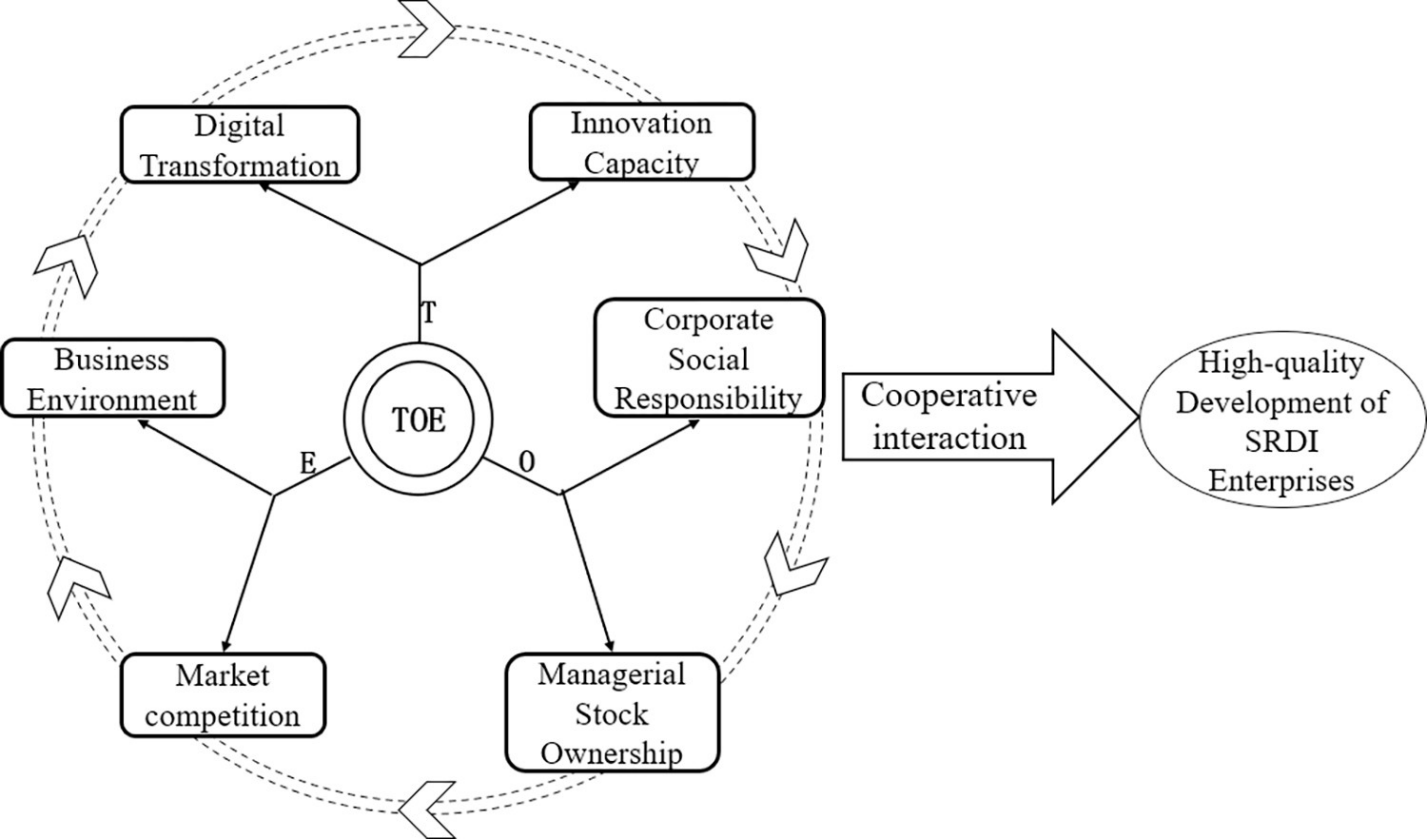
Theoretical framework of SRDI enterprises’ high-quality development.

The above analysis highlights the impact of six antecedent elements within the TOE framework on the high-quality development of SRDI enterprises. However, these elements are interdependent, functioning in a synergistic manner. Innovation capability is crucial for digital transformation and digital technology application. Additionally, management shareholding enhances decision-making accuracy, while corporate social responsibility is key to adapting to industry competition and the business environment [52]. The interaction among the TOE framework’s six elements significantly boosts SRDI enterprises’ specialization, refinement, distinctiveness, and innovation, fueling high-quality development in challenging scenarios. This study presents a model for SRDI enterprises’ high-quality development, driven by six key factors: innovation capability, digital transformation, corporate social responsibility, management shareholding, market competition, and business environment, illustrated in Fig 1.

## 4. Research design

### 4.1 Research method

The QCA method is a set analysis method based on set theory, developed by Professor Ragin, an American sociologist, in 1987. This method posits that the impact of variables on outcomes is not independent; rather, the effect of a variable is contingent upon its combination with other variables. The QCA method employs set theory to transform antecedent condition variables and outcome variables into sets for analysis, focusing on the subsets within. Specifically, the QCA method first selects appropriate calibration standards based on existing theories or empirical knowledge, aligning the conceptualized antecedent condition variables and outcome variables with case set memberships. Subsequently, it performs sufficiency, necessity, and counterfactual analyses on the antecedent conditions or their combinations. This approach elucidates the complex causal relationships between antecedent conditions or their combinations and the outcome variables [53]. Analyzing the set relationships between condition sets and outcome sets, grounded in set theory, is particularly apt for social science research. This is because social science research data, primarily linguistic, are challenging to quantify yet can be formally articulated as relationships between sets [53]. For instance, in social science research, the proposition “Start-up companies are highly innovative” can be framed as a set relationship: “Start-up companies (set) constitute a subset of highly innovative enterprises (set).” Unlike traditional correlation analysis techniques such as multiple regression analysis and SEM, the QCA method does not merely explore variable correlations but delves into how “Entrepreneurship relates to innovation.” Additionally, set relationships are categorized into two fundamental types: definitional and causal. Definitional set relationships typically articulate simple definitions, for example, “An apple is a fruit.” Causal set relationships elucidate whether a set of antecedent conditions causes the outcome set or explain the connections between sets. QCA analysis pertains to examining causal set relationships [53].

The QCA method, emerging as a social science research paradigm that transcends the limitations of traditional quantitative and qualitative approaches, has been extensively applied in management studies [54]. This study adopts a configurational perspective, utilizing the fsQCA method to investigate how six antecedent elements: digital transformation, technological innovation, corporate social responsibility, managerial stock ownership, market competition, and business environment, interactively influence the development of SRDI enterprises. The primary reasons for selecting this method include: (1) The QCA method enables the exploration of “joint effects” and “interactive relationships” between antecedent elements of a specific phenomenon, aligning closely with this study’s goal to uncover the complex mechanisms driving the high-quality development of Chinese SRDI enterprises [53]. (2) Focusing on 141 SRDI “little giant” enterprises, the limited sample size challenges the attainment of robust results through traditional statistical methods. In comparison, the QCA method proves more apt for this study than traditional statistical analysis methods. Furthermore, while cluster and factor analysis can assess configurational relationships, they fall short in identifying conditionality dependence, configurational equivalence, and causal asymmetry. (3) Fuzzy-set Qualitative Comparative Analysis (fsQCA) excels in capturing the nuanced effects of antecedent elements, offering superior handling and analysis of continuous variables with greater data precision compared to other QCA techniques such as csQCA and mvQCA.

### 4.2 Data selection

This study selects 141 Chinese national-level SRDI “little giant” listed companies in 2020 as research samples, based on the availability of corporate social responsibility and business environment data. To ensure a dynamic and comprehensive analysis, this study calculates the Total Factor Productivity (TFP) using data from 2020 and 2021 for the sample companies. Data on corporate social responsibility and city business environment are sourced from Hexun.com and the “2020 China City Business Environment Evaluation”, respectively. All other antecedent variable data are obtained from the CSMAR database.

### 4.3 Variable design

#### 4.3.1 Outcome variables

Total factor productivity (TFP) measures the efficiency of factor utilization in organizations, reflecting the high-quality development of enterprises and their resource allocation efficiency. Drawing on prior research, this study adopts TFP as a proxy for SRDI enterprises’ high-quality development [55]. The Levinsohn-Petrin (LP) method is used to calculate TFP, assessing SRDI enterprises’ development quality.

#### 4.3.2 Conditional variables

Innovation capacity. The quality of technological innovation is crucial for SRDI enterprises to overcome critical technological challenges. This study measures innovation capability by the citation count of invention and utility model patents, reflecting patent value and innovation quality. This study adds one to the citation count of invention and utility model patents and uses the natural logarithm as the innovation capability metric [56].

Digital transformation. Adopting methods from prior research [57], this study quantifies enterprises’ digital transformation levels by analyzing the frequency of specific keywords in their annual reports. Keywords for digital transformation include five categories: artificial intelligence, blockchain, cloud computing, big data, and digital technology application. The study calculates the total frequency of these keywords in the annual reports of sample companies. Then, it uses the logarithm of this total frequency plus one as a proxy indicator for digital transformation.

Corporate social responsibility. This study utilizes the 2020 corporate social responsibility scores from China’s Hexun.com, published in 2021, as indicators of CSR performance, in line with existing research [58]. Hexun.com’s CSR rating system comprises 56 indicators, with higher scores indicating better CSR performance.

Managerial stock ownership. In line with existing literature, this study measures management shareholding levels by the ratio of shares held by management to the total shares [59].

Market competition. Following existing literature, this study quantifies market competition intensity through the absolute value of the Herfindahl Index’s logarithm [60].

Business environment. Following existing research methods, this study uses the “2020 China City Business Environment Evaluation” to measure the business environment scores of the cities hosting the sample enterprises [61].

### 4.4 Data calibration

Data calibration is the process of assigning set membership scores to cases, thereby transforming them into fuzzy sets [54]. Research into the complex causal relationships of SRDI enterprises’ high-quality development is nascent, with a lack of external guidance and theoretical foundation for anchor setting. Drawing on existing research, this study uses the direct calibration method with 95%, 50%, and 5% percentiles as anchors for both outcome and antecedent variables, based on sample case conditions and data traits [62]. The calibration anchors of variables and their descriptive statistics are detailed in Table 1.

**Table 1:** Descriptive statistics and calibration anchor points.

Conditional Variable	Descriptive statistics				Calibration anchor points		
	Max	Min	Mean	SD	Full Affiliation	Intersection Point	Completely Unaffiliated
TFP	15.951	12.613	14.120	0.642	15.415	14.061	13.311
IC	752.000	0.000	52.971	116.218	313.800	5.000	0.000
DT	1199.000	0.000	60.021	142.840	316.200	8.000	0.000
CSR	36.480	-3.150	21.711	7.917	30.623	23.450	0.000
MSO	94.000	0.000	30.281	21.408	64.341	31.109	0.000
MC	3.335	0.144	2.362	0.602	3.320	2.453	1.415
BE	73.301	7.322	37.790	22.461	73.301	31.755	10.576

## 5. Empirical analysis

### 5.1. Necessary condition analysis

Following the fsQCA method’s general steps, it’s essential to first analyze the “necessity” of antecedent conditions and outcome variables before a “sufficiency” analysis. A necessary condition is an antecedent always present with the outcome, judged by the consistency level between the antecedent and the outcome variable. If an antecedent condition’s consistency level exceeds 0.9, it is deemed necessary for the outcome variable [53]. Table 2 presents the fsQCA3.0 software-derived necessity analysis results for high-quality/non-high-quality development.

**Table 2:** Analysis of necessary conditions.

Conditional Variable	High-quality development		Conditional Variable	Non-high-quality development	
	Consistency	Coverage		Consistency	Coverage
IC	0.591	0.724	~IC	0.804	0.693
DT	0.569	0.671	~DT	0.757	0.669
CSR	0.754	0.641	~CSR	0.632	0.747
MSO	0.613	0.594	~MSO	0.635	0.653
MC	0.687	0.667	~MC	0.702	0.721
BE	0.629	0.603	~BE	0.639	0.665

In Table 2, the abbreviations are defined as follows: IC: High innovation capability, ~IC: Non-high innovation capability. DT: High digital transformation, ~DT: Non-high digital transformation. CSR: High corporate social responsibility performance, ~CSR: Non-high CSR performance. MSO: High management shareholding, ~MSO: Non-high management shareholding. MC: High market competition, ~MC: Non-high market competition. BE: Superior business environment, ~BE: Non-superior business environment.

Table 2 reveals that all antecedent condition variables’ consistency levels with the outcome variable fall below 0.9, indicating no necessary conditions for SRDI enterprises’ high-quality/non-high-quality development. The weak explanatory power of individual antecedent variables on SRDI enterprises’ high-quality/non-high-quality development suggests the complexity of influencing factors. This complexity means that a single factor cannot fully explain SRDI enterprises’ high-quality development, necessitating further configuration analysis.

### 5.2 Conditional configuration analysis

The aim of configuration analysis is to uncover how various combinations of antecedent conditions sufficiently lead to outcomes. Based on existing research [63] and sample data characteristics, this study establishes a case frequency threshold of 1, an original consistency threshold of 0.8, and a PRI consistency threshold of 0.65. The fsQCA3.0 software’s sufficiency analysis generates complex, parsimonious, and intermediate solutions. In line with existing research [63], this study focuses on intermediate solutions to report sufficiency condition results and employs parsimonious solutions to differentiate core from peripheral conditions. Table 3 presents the results of analyzing six antecedent conditions for SRDI enterprises’ high-quality development through configuration analysis.

**Table 3:** Configurations for achieving high-quality development.

Parameterization	High-quality development			
	H1	H2	H3	H4
IC	•	●	●	●
DT	●	●	⊗	•
CSR	⊗	●		●
MSO	●	⊗	●	●
MC		⊗	●	
BE	⊗	⊗	⊗	●
Consistency	0.939	0.948	0.949	0.904
Raw coverage	0.205	0.203	0.224	0.218
Unique coverage	0.010	0.044	0.050	0.062
Solution consistency	0.905			
Solution coverage	0.372			

Note: referring to Fiss’s labeling method, “●” indicates the presence of core conditions; “⊗” indicates absence of core conditions; “●” indicates the presence of an auxiliary condition; “⊗” indicates absence of an auxiliary condition; blank indicates that this condition is optional. The following table is the same.

#### 5.2.1 Analysis of configurations with high-quality development

Table 3 reveals four configurations that lead SRDI enterprises to high-quality development, considered sufficient condition combinations. The consistency levels of these configurations exceed the acceptable threshold of 0.80, indicating their sufficiency for SRDI enterprises’ high-quality development. With an overall consistency level of 0.905, 90.5% of SRDI enterprises matching these configurations have attained high-quality development. Furthermore, the model’s overall coverage of 0.372 suggests it explains 37.2% of SRDI enterprises’ high-quality development cases. Despite the model’s relatively low coverage of 0.372, it’s deemed acceptable given the study’s sample size of 141, surpassing the typical range for QCA methods (15–80). The study’s results are promising, with the four configurations effectively elucidating SRDI enterprises’ high-quality development. We will now proceed to analyze each of these four configurations in detail.

Configuration H1 (IC*DT*~CSR*MSO*~BE) shows that SRDI enterprises, with high levels of digital transformation and managerial stock ownership, can achieve high-quality development despite lower corporate social responsibility performance and a poor business environment. H1 accounts for about 20.5% of the case companies. In a poor business environment, low corporate social responsibility hinders SRDI enterprises from enhancing their reputation and expanding into niche markets. Managerial stock ownership helps SRDI enterprises mitigate the principal-agent problem, encourages management to make digital-age decisions, and boosts business empowerment through digital transformation. SRDI enterprises can modernize production tools with digital technology for automated and intelligent processes, enhance inter-enterprise information sharing, improve management systems for better departmental collaboration, and boost operational efficiency, thus forging a core competitive advantage and achieving high-quality development. This result supports Wu et al. and Chen’s [21, 46] findings, underscoring the positive impact of technological innovation, digital technology, and management efficiency on enterprise development. For instance, SFZN Company, based in ** City, Hubei Province, is a national high-tech enterprise specializing in intelligent control technology. With years of specialization, SFZN has increased managerial stock ownership to address the principal-agent problem, leading to management decisions favoring digital transformation and the application of digital technology in its operations. Digital technology empowers all aspects of SFZN’s operations. Digital technology has enhanced SFZN’s production intelligence, communication efficiency, and departmental collaboration, improving management and operational efficiency. Despite ** City’s challenging business environment, SFZN has become a leader in intelligent logistics equipment with advanced technology and a wide range of products.

Configuration H2 (IC*DT*CSR*~MSO*~MC*~BE) shows that SRDI enterprises can achieve high-quality development through technological innovation, digital transformation, and fulfilling corporate social responsibility, even with lower levels of managerial stock ownership, market competition, and a weaker business environment. This path accounts for about 20.3% of the case companies. Under poor business environments, with low managerial stock ownership and market competition, SRDI enterprises can boost their use of digital technology through digital transformation. Digital technology accelerates knowledge and technology flow, boosts external knowledge acquisition and integration, and enhances technological innovation in enterprises. Fulfilling corporate social responsibility enhances the external network, strengthens digital transformation’s impact on innovation [64], meets evolving market needs, boosts competitiveness, and achieves high-quality development. This finding underscores the critical role of technological innovation, digital technology, and corporate social responsibility in SRDI enterprises’ high-quality development, aligning with Cheng et al. and Xue et al.’s [19, 25] conclusions. Using YYDQ Company as an example, located in ** city, Jiangsu Province, it is a high-tech enterprise focused on R&D, manufacturing, and selling core automotive electronics. It specializes in vehicle intelligent power controllers. Actively engaging in digital transformation, the company accelerates knowledge and technology flow, boosting its innovation and integration capabilities. Simultaneously, prioritizing customer satisfaction and enhancing corporate social responsibility, the company builds a strong reputation and a robust external network, facilitating resource flow. Gaining a competitive edge in its niche market has enhanced the company’s development quality. With over 500 patents from independent R&D, the company is now a global leader in vehicle rectifiers and regulators, boasting an annual revenue of 1.2 billion yuan and improved development quality.

Comparing configurations H1 and H2 reveals a lack of environmental conditions; however, technical and organizational conditions alone can propel SRDI enterprises towards high-quality development. Hence, this study designates configurations H1 and H2 as the “T-O self-driven” pathway for SRDI enterprises’ high-quality development.

Configuration H3 (IC*~DT*MSO*MC*~BE) shows that without digital transformation and a supportive business environment, market competition encourages SRDI enterprises to pursue high-quality development via technological innovation and higher managerial stock ownership. About 22.4% of the case companies align with Configuration H3. In suboptimal business environments, intense market competition motivates SRDI enterprises to boost managerial stock ownership, thereby spurring management’s drive for technological innovation. Technological innovation enhances enterprises’ analytical, predictive, and sales capabilities in marketing, allowing for precise market demand response and improved marketing effectiveness [34]. This, in turn, boosts total factor productivity and fosters high-quality development in SRDI enterprises. For instance, HQGF Company, situated in ** County, Guangdong Province, specializes in R&D, production, and sales of concrete admixtures, faces a challenging business environment and intense market competition. Intense competition has led the company to increase managerial stock ownership, fostering greater openness to technological innovation among management. Its technological innovation activities enable precise market analysis and predictions, customer-centric approaches in niche markets, dynamic resource allocation, and process transformation to enhance customer satisfaction through refined management. The company has invested heavily in technological innovation, establishing top-tier R&D and engineering centers for additives and concrete. It has also increased managerial stock ownership to improve management and decision-making, and partnered with universities like Tsinghua for integrated production, education, and research efforts. Currently, HQGF ranks among the top three in the nation in terms of comprehensive strength, with annual sales exceeding 1.33 billion yuan, marking a significant development milestone.

Configuration H4 (IC*DT*CSR*MSO*BE) demonstrates that a comprehensive drive encompassing technology, organization, and environment facilitates high-quality development in enterprises. SRDI enterprises, situated in a favorable business environment and endowed with technological innovation capabilities, a commitment to corporate social responsibility, and significant managerial stock ownership, can attain high-quality development when augmented by digital technology. H4 can explain 21.8% of the case companies. Research has discovered that an efficient and perfect match between internal resources and external conditions significantly enhances enterprise development quality [65]. A premium business environment offers SRDI enterprises an excellent external institutional backdrop to overcome the specialization “lock-in” dilemma; in such an environment, SRDI enterprises with considerable managerial stock ownership are inclined to make decisions that serve stakeholders’ interests. These include fostering a positive corporate reputation through active corporate social responsibility and boosting core competitiveness through technological innovation, thereby advancing the enterprise’s sustainable development and high-quality growth. For instance, RTJK company, located in ** city and specializing in the development, design, manufacturing, and sales of fashion health appliances, epitomizes this approach. The company leverages a superior business environment, managerial stock ownership for decision-making precision, a strong commitment to corporate social responsibility, and continuous technological innovation, securing over 430 patents and establishing a unique competitive edge. Its products, celebrated nationwide and globally, have penetrated markets in Southeast Asia, the Middle East, Europe, and North America, marking significant developmental milestones.

Upon comparing configurations H3 and H4, it is observed that both encompass technology, organization, and environmental conditions, highlighting the joint influence of technology, organization, and environmental conditions in propelling SRDI enterprises towards high-quality development. Thus, this study proposes the term “T-O-E Synergistic Drive” for the pathways H3 and H4, reflecting their role in fostering high-quality development in SRDI enterprises.

#### 5.2.2 Configuration analysis of non-high-quality development

Based on the asymmetry of the causality of the QCA method, this study analyses the non-quality development groupings of the sample firms, as shown in Table 4. Table 4 reveals four configurations (NH1, NH2, NH3, NH4) delineating the non-high-quality development paths of SRDI enterprises. All four configurations exceed the acceptable consistency threshold of 0.80, serving as sufficient conditions for the non-high-quality development of SRDI enterprises. With an overall consistency level of 0.819, these configurations account for 81.9% of SRDI enterprises in the sample achieving non-high-quality development. Additionally, the model’s solution coverage is 0.639, explaining 63.9% of SRDI enterprises’ non-high-quality development cases. Next, we will analyze these four configurations individually.

**Table 4:** Configurations for non-high-quality development.

Parameterization	Non-high-quality development			
	NH1	NH2	NH3	NH4
IC	⊗	⊗	⊗	⊗
DT	⊗		⊗	•
CSR		⊗		⊗
MSO		•	●	⊗
MC	⊗			●
BE		●	●	●
Consistency	0.838	0.852	0.875	0.891
Raw coverage	0.528	0.296	0.317	0.233
Unique coverage	0.199	0.018	0.010	0.038
Solution consistency	0.819			
Solution coverage	0.639			

Configuration NH1(~IC*~DT*~MC) reveals that SRDI enterprises struggle to achieve high-quality development in the absence of innovation capability, digital transformation, and market competition as core conditions, regardless of the business environment, corporate social responsibility fulfillment, or management stock ownership. The absence of technological innovation hampers enterprises’ market competitiveness and ability to introduce new products, while a low level of digital transformation reflects poor application of digital technology, customer interaction, data management, and reduced efficiency in resource allocation and management. Insufficient market competition can deprive enterprises of external stimuli, weakening the drive for technological innovation and digital transformation, and hindering high-quality development.

Configuration NH2(~IC*~CSR*MSO*BE) illustrates that lacking innovation and corporate social responsibility, even with a favorable business environment and management shareholding, impedes SRDI enterprises from reaching high-quality development. Although a favorable business environment lays the groundwork for SRDI enterprises, the absence of innovation and poor performance in corporate social responsibility hinder achieving high-quality development.

Configuration NH3 (~IC*~DT*MSO*BE) reveals that without innovation capability and digital transformation, even with a supportive business environment and management shareholding, SRDI enterprises struggle to achieve high-quality development. Innovation capability serves as the soul and cornerstone of high-quality development for SRDI enterprises. The presence of management shareholding and a favorable business environment cannot offset the absence of innovation capability, hindering high-quality development.

Configuration NH4 (~IC*DT*~CSR*~MSO*MC*BE) indicates that the absence of innovation capability, corporate social responsibility, and management shareholding, despite the presence of digital transformation, market competition, and a favorable business environment, makes high-quality development challenging for SRDI enterprises. The essence of high-quality development for SRDI enterprises lies in leveraging their core strengths rather than solely depending on external factors like market competition and the business environment. When there are obvious deficiencies in the enterprises’ own technical and organisational conditions, it is difficult for SRDI enterprises to achieve high-quality development even if the impetus of external environmental factors is strong.

A comparison of high versus non-high-quality development paths reveals that innovation capability is prevalent in high-quality configurations but notably absent in non-high-quality ones, underscoring its significance for SRDI enterprises’ high-quality development. An analysis of environmental factors across different development configurations shows that while a positive business environment supports high-quality development for SRDI enterprises, this is contingent upon the enterprises’ superior innovation and organizational capabilities. Even with a favorable business environment, SRDI enterprises struggle to achieve high-quality development if their capabilities are lacking.

### 5.3 Robustness analysis

(1) A robustness test was conducted by adjusting the calibration anchor points from “95%, 50%, and 5%” to “80%, 50%, and 20%”. The adjusted configurations of high-quality development, as shown in Table 5, reveal that configurations H1, H2, and H3 stayed unchanged. For H4, digital transformation ascended to a core condition, and market competition’s requirement was modified, allowing for its presence or absence—maintaining consistency with the initial scenario. This demonstrates the research conclusions’ relative robustness.

**Table 5:** Robust test of configurations for high-quality development.

Parameterization	High-quality development			
	H1’	H2’	H3’	H4’
IC	•	●	●	●
DT	●	●	⊗	●
CSR	⊗	●		●
MSO	●	⊗	●	•
MC		⊗	●	
BE	⊗	⊗	⊗	●
Consistency	0.939	0.904	0.949	0.949
Raw coverage	0.205	0.218	0.203	0.224
Unique coverage	0.010	0.062	0.045	0.050
Solution consistency	0.878			
Solution coverage	0.305			

(2) Further affirming the reliability of these findings, the consistency threshold was raised from 0.8 to 0.85 [61]. Post-adjustment, as detailed in Table 6, neither the configurations, case numbers, solution consistency, nor coverage altered, underscoring the robustness of the research conclusions.

**Table 6:** Robust test of configurations for high-quality development.

Parameterization	High-quality development			
	H1”	H2”	H3”	H4”
IC	•	●	●	●
DT	●	●	⊗	•
CSR	⊗	●		●
MSO	●	⊗	●	●
MC		⊗	●	⊗
BE	⊗	⊗	⊗	●
Consistency	0.939	0.948	0.949	0.904
Raw coverage	0.205	0.203	0.224	0.218
Unique coverage	0.010	0.044	0.050	0.062
Solution consistency	0.905			
Solution coverage	0.372			

## 6.Conclusions and policy implications

### 6.1 Conclusions

This study utilizes the TOE theoretical framework and fsQCA method to analyze the impact of six factors—technological innovation, digital transformation, corporate social responsibility, managerial stock ownership, market competition, and business environment—on the high-quality development of 141 national SRDI “little giant” enterprises in China. The study reveals that (1) technological, organizational, and environmental factors are not sufficient on their own for SRDI enterprises to achieve high-quality development. (2) Two paths exist for SRDI enterprises’ high-quality development: “T-O self-driven” and “T-O-E Synergistic Drive”. Additionally, four paths lead to the non-high-quality development of SRDI enterprises. The synergistic interplay of multiple factors, which can combine in various “equivalent” ways, drives the high-quality development of SRDI enterprises. (3) Technological innovation universally influences SRDI enterprises’ paths to high-quality development. It serves as a crucial breakthrough for their growth and a key solution to China’s “stuck neck” technology challenges.

### 6.2 Policy implications

This study investigates the relationship between technology, organization, environmental conditions, and high-quality enterprise development. It encourages SRDI enterprises to adopt a holistic approach, considering the current market competition and business environment, to achieve synergistic integration across technology, organization, and environment, focusing on optimizing the combination of technological innovation and other precursor conditions. Specifically, this study offers two policy recommendations for SRDI enterprises aiming at high-quality development.

At the enterprise level, SRDI enterprises should pursue innovation-driven development, accelerate breakthroughs in key core technologies, and aim for high-end upgrades. Firstly, SRDI enterprises should boost their R&D investment, increase its share of operating income, and enhance their technological innovation capabilities. Simultaneously, SRDI enterprises should optimize their use of innovation resources, leveraging national and local platforms, funds, and rewards, and enhance collaboration with universities, research institutions, and industry associations to share and synergize innovation resources. Secondly, SRDI enterprises should enhance the organization and management of innovation, developing incentive and evaluation mechanisms, fostering an innovation culture, and stimulating motivation and vitality. Additionally, SRDI enterprises should better monitor and evaluate innovation efficiency, protect and commercialize innovation outcomes, and promote their rapid application to maximize benefits. Lastly, SRDI enterprises should broaden and deepen their innovation efforts, not just in niche markets and core products but also across industry and value chains, aiming to meet existing needs and anticipate future demands, thereby enhancing innovation quality and level.

At the government level, the government is tasked with formulating and implementing policies that foster a conducive policy and business environment, thereby facilitating the high-quality growth of SRDI enterprises. Initially, the government should refine the policy framework for SRDI enterprises by developing specific regulations, measures, and standards. Concurrently, the government must enhance policy supervision, ensure effective implementation, and timely adjust policies to maintain their relevance and efficacy. Secondly, the government should diversify its support for SRDI enterprises through financial subsidies, tax breaks, loans, credit guarantees, equity investments, and enhanced public services. Finally, the government should actively optimize the business environment to provide SRDI enterprises with favorable external conditions, thus enhancing their development potential and promoting sustained growth. This includes improving the legal framework to protect SRDI enterprises’ rights, ensuring market order, and fostering fair competition, as well as breaking market barriers and expanding market access to enhance market participation of SRDI enterprises.

### 6.3 Research limitations and prospects

This study’s proposed analysis, based on the TOE framework, of the synergistic development path for SRDI enterprises’ quality, presents limitations and areas for enhancement that merit further exploration: First, the technological, organizational, and environmental factors influencing SRDI enterprises’ development evolve as the enterprise life cycle advances. However, this study, relying on cross-sectional data, overlooks the dynamic evolution of technological, organizational, and environmental factors. Future research should consider the time-based dynamics of technological, organizational, and environmental elements. By employing dynamic QCA methods for time-sequential qualitative comparative analysis, it could explore the intricate effects of these conditions on SRDI enterprises’ high-quality development during dynamic changes, aiming to enhance case configuration coverage and validity. Second, the focus on SRDI “little giant” listed companies as samples—while most SRDI enterprises are not listed—partially limits the conclusions’ generalizability. Future studies could broaden the research conclusions’ applicability by collecting data from non-listed SRDI enterprises through surveys, investigating paths towards high-quality development.

## Supporting information

S1 Data (CSV)
